# Health workers’ perceptions of private-not-for-profit health facilities’ organizational culture and its influence on retention in Uganda

**DOI:** 10.1186/s12913-017-2763-5

**Published:** 2017-12-06

**Authors:** Constance Sibongile Shumba, Karina Kielmann, Sophie Witter

**Affiliations:** Queen Margaret University, Queen Margaret University Drive, Musselburgh, East Lothian EH21 6UU UK

**Keywords:** Health workers’ retention, Organizational culture, Private-not-for-profit health facilities, Uganda

## Abstract

**Background:**

An in-depth understanding of how organizational culture is experienced by health workers (HWs), and influences their decisions to leave their jobs is a fundamental, yet under-examined, basis for forming effective retention strategies. This research examined HWs’ working experiences and perceptions of organisational culture within private-not-for-profit, largely mission-based hospitals, and how this influenced retention.

**Methods:**

Thirty-two HWs, including managers, in 19 health facilities in Uganda were interviewed using a semi-structured topic guide. Interview transcripts were analysed using thematic content analysis.

**Results:**

Interviews showed that the organizational culture was predominantly hierarchical, with non-participative management styles which emphasized control and efficiency. HWs and managers held different perceptions of the organizational culture. While the managers valued results and performance, HWs valued team work, recognition and participative management.

**Conclusions:**

The findings of this study indicate that organizational culture influences retention of HWs in health facilities and provide a useful context to inform health care managers in the PNFP sub-sector in Uganda and similar contexts. To improve retention of HWs, a gradual shift in organizational culture will be necessary, focussing on the values, beliefs and perceptions which have the greatest influence on observable behaviour.

## Background

Uganda is among the 57 countries that was identified in 2006 as having a critical shortage of Health workers (HWs) with health worker numbers and skill mix deemed inadequate for service provision [1]. The country has severe shortage of HWs with a ratio of about 1.55 HWs per 1000 population, which is far below the minimum World Health Organization prescribed number of 2.28 HWs (doctors, nurses and midwives only) per 1000 population [2, 3]. Staffing establishments in PNFP hospitals are required to follow the minimum-staffing norms set by the Government, although they can be adjusted to cater for variations in local conditions [4]. In the country, both the public sector and private-not-for-profit (PNFP) health facilities are unable to retain HWs [5] and while retention of HWs is a major problem for the country at large, the magnitude is greater in the PNFP sector compared to the public sector [6]. In 2013/2014 when local governments recruited HWs across the country, 25% of the recruits came from the PNFP sector [6] and some faith-based PNFP health facilities lost almost three-quarters of their HWs to the public sector [7]. The PNFP sub-sector is an integral part of the Ugandan health system, offering health services mostly in remote, rural, hard-to-reach areas. The PNFP sub-sector employs about 35% of the country’s HWs and more than 40% of the health sector outputs are derived from it [6, 8]. The PNFP sub-sector also owns nearly three quarters of health training institutions, with 65% of the country’s midwives and nurses trained from PNFP health training institutions [4–9]. Some of the improvements in health indicators in the country are attributed to the role of PNFPs in health service delivery and training of health workers.

### Organizational culture

Organizational culture is *“the pattern of shared values and beliefs that help individuals understand organizational functioning and thus provide them with norms for behaviour in the organization”* [10]. The values and assumptions are engrained in the organization and may evolve slowly over time [11]. Differences in retention among organizations could be linked to the cultural values that influence human resource strategies including human resource practices. Organizational cultural values influence the management systems, and are also reinforced by the human resource practices and behaviours [12]. These practices influence perceptions of HWs’ towards health facilities’ decision-making, leadership and work environment that affect the commitment and retention of employees [12–15]. In this study, organizational culture refers to the way things are done in the organization, as influenced by the common values and assumptions.

Generally, the literature posits two main frameworks on organizational culture. The first tends to classify cultures into two main groups: flexible or rigid [13]. The Competing Values Framework (CVF) suggests that there are four archetypes within these two distinct groups, namely: human relations culture; entrepreneurial culture; hierarchical culture; and market culture [16]. Flexible cultures have been associated with higher job satisfaction specifically, the human relations culture, which is about teamwork, employee empowerment and participation and entrepreneurial culture, which is about creativity, innovation and change. Rigid cultures include hierarchical culture which stresses bureaucratic and centralized structure and rules, with limited autonomy, and market culture, which is geared towards competitiveness, efficiency and productivity.

The second framework is based on Schein’s (1992) layers of organisational culture, namely; artefacts which are the organizational structures and processes, values which are the strategies, goals and philosophies, and basic assumptions, which are the unconscious beliefs and perceptions [17]. Organizational culture exists at the level of the organization’s visible structures which in turn influence observable behaviour, including decision-making [18]. The visible structures can be superficial in comparison to the values and basic assumptions, which have greater depth and influence visible behaviour. A quantitative study among physicians working in group practice settings in the United States found that organizational culture affected physician job satisfaction [19]. A quantitative study conducted among hospital nurses in Taiwan found a positive correlation between organizational culture with leadership behaviour and job satisfaction [20].

### Organizational culture and the PNFP sub-sector in Uganda

While most PNFP health facilities are faith-based, some non-faith based PNFP health facilities are owned by humanitarian and community based organizations. PNFP health facilities’ working conditions are different from those in the government facilities with greater workload, supervision and control by managers [5, 10]. It has been previously reported that managers believe that HWs who join the faith-based PNFP health facilities are aware of the workload and therefore expected to adjust as appropriate [7]. The PNFP sector in Africa is known to require more commitment in comparison to the public sector [21]. However, faith based PNFP health facilities have been reported to have undemocratic tendencies [7]. A cross-sectional survey conducted among 127 health care workers in Mahalapye and Ngamiland districts of Botswana found that HWs preferred organisational values of transparency, professional growth, staff recognition, shared decision-making, accountability, productivity, leadership development and teamwork and the lack of these resulted in negative experiences and low motivation [22].

This qualitative study explored the organizational culture in PNFPs, largely mission-based health facilities, from the perspective of both health workers and managers. There have been no previous studies exploring the link between organizational culture and retention in the PNFP sub-sector in Uganda, and there are not many across sub-Saharan Africa [22, 23]. We examined as part of a broader study, the experiences of 32 HWs working in PNFP health facilities and how these influence retention decisions. The influence of organizational culture on retention emerged as one of the major themes from this broader study, adding new knowledge in this field internationally.

## Methods

The study was conducted in 19 PNFP health facilities geographically spread across Uganda in west, north, east and central regions. The philosophical perspective of this research was an interpretive paradigm and the research followed a phenomenological descriptive approach, to understand individual HWs’ experiences. A total of 32 semi-structured face-to-face interviews were held with 23 HWs and five managers who were employed in the health facilities at the time of the study, and four HWs who were previously employed in the health facilities who volunteered to participate in the study.

### Data collection

Data was collected over a period of 8 months, starting 1st August 2013 to 3rd March 2014. The interviews were held with doctors, clinical officers/medical assistants, nurses, laboratory technicians and managers to understand their views and experience on working in the PNFP health facilities. Semi-structured interview guides for both HWs and managers were developed and refined based on a literature review. Probing was used to follow-up on any ideas expressed earlier in the interview. The semi-structured interview guides were pilot tested among 5 HWs at a small health facility in Kampala and led to some rephrasing of questions to improve their clarity. All interviews were conducted with the backing of audio tape recordings and augmented with the note-taking and transcribed.

### Sampling

The health facilities were purposively chosen because they are part of the PNFP health sub-sector and provided a good mix of faith-based (15) and non-faith based (four) health facilities in the PNFP health sub-sector, covering the four main regions of the country. Two of the 19 health facilities were Protestant facilities affiliated to the Church of Uganda (Anglican), while the 13 facilities were affiliated to the Catholic Church. The majority of the PNFP health facilities were hospitals located in rural and hard to reach areas and those in urban areas targeting the urban poor. Almost all the facilities were hospitals with bed capacities ranging from 0 to 540, except two that were stand-alone HIV treatment centres. Purposive sampling was used to recruit the volunteers among the different cadres of HWs cadres with at least 6 months duration working in the health facility. Five HWs who had left the study health facilities were identified through snowball sampling, where a key participant was identified and recommended other four information-rich cases who could contribute to this research.

### Data processing and analysis

The researchers transcribed all data verbatim and referred to field notes for non-verbal communication, including body language, facial expressions and tone of voice. Thematic content analysis was done manually. The transcripts were read and reviewed on several occasions by one of the researchers, who identified themes based on the questions. The themes formed the basis of a coding system which allowed the researcher to organize the data in tabular form. The analytic framework was primarily based on a combination of the original set of questions and emergent themes. Several dimensions of organizational culture were explored, mainly management style, teamwork, communication, workload, autonomy and extent of internal or external orientation.

### Ethical considerations

Ethical approval was obtained from the Queen Margaret University Research Ethics Panel, the Uganda National Council for Science and Technology (SS 3193) and Lacor Hospital Institutional Review Board (LHIREC 048/11/13). Relevant permissions were also granted by the Catholic and Protestant Medical Bureaus, as well as the participating health facilities. Interviews were done in places that allowed for privacy as agreed with participants. All participants were informed that their participation in the study was voluntary, and that they could refuse to answer any questions and could choose to withdraw from the study at any time. Written informed consent was obtained prior to the interviews. Permission to audio-record interviews was also obtained from the participants. All identifiers were removed from the transcripts.

### Characteristics of participants and health facilities

Study participants had been at their current facilities for periods ranging from 7 months to 10 years, with most of them having worked in the study facilities for more than 1 year. Twenty respondents were male, and 12 were female. In some cases, the respondent had a dual role as frontline HW and as manager, the managerial role was considered when interviewing them. The average age was 30 years, with a minimum age of 21, while the maximum one was 68 years. The minimum education level was a certificate, while the maximum was a masters’ degree. There were nine doctors, six clinical officers and six nurses, six laboratory technologists and five managers. Nine of the respondents were from the northern region, 12 from the western region, two from the eastern region and nine from the central region.

## Results

We first discuss the experiences of HWs regarding the work environment and work culture as part of the layer of artefacts or the organization’s visible structures which influence the observable behaviour of HWs and their retention decisions. The first key area of results relates to organizational influences that enable HWs to self-actualize by providing a good work environment, tools and varied tasks. Relational dynamics also contribute to the organizational culture in PNFPs with HWs placing value on their peers’ support to facilitate their decisions to stay. We then present their subsequent working experiences, unravelling the values and basic assumptions. The second area of results concerns the professional and social divides that characterise health workers’ reported experiences of working in PNFP health facilities.

### Work environment and work culture

The importance of the work environment to HWs was highlighted in the findings and is consistent with the organizational structures and processes dimension of organizational culture. HWs valued professionalism, self-actualization and good interpersonal relationships. On the other hand, high workload and limited focus on staff welfare negatively influenced motivation of HWs.

### Professionalism and self-actualisation

HWs explained that they were motivated by environments where there are more challenging tasks, as well as opportunities for diverse roles in different departments. Participants explained that they are attracted to work and stay in these PNFP health facilities because they could rotate through different departments and get varied experience. This was particularly true for HWs who had just completed their studies. Others explained that they wanted to work in environments where they could utilize their skills, where they are able to put in practice what they have studied.

*[…] the most important thing is what kind of environment I am going to work in, that to me is very important. I want to work in an environment where I will have to do what I studied. For example, not only seeing HIV patients but doing comprehensive medicine whereby I do both medicine and surgery so that is something very important to me. […] Male* Doctor and manager, western Uganda R15

*For me it’s the experience I am getting. It’s good. we have the HIV clinic, we have the family planning unit, at least I am benefiting a lot, and right now I know more about HIV, about ARVs and this is good for me […]*. Male Clinical officer, northern region R18

HWs explained that a conducive working environment with the necessary provisions to do their professional work was more satisfying and influenced them to stay in the PNFP organizations:

*I am motivated by how well a patient gets…I do not want to work in a setting where I prescribe, but there are no drugs when I see a patient is in pain or is ill. I mean we have never run out of drugs, not even a single day you send a patient out without drugs. […] The government hospitals do not have adequate facilities.* Male clinical officer, northern Uganda R1

### Good interpersonal relationships- *“the psychological contract”*

As part of the work environment, HWs explained that team work, where the workers had good interpersonal relationships and solidarity even when the managers and supervisors were not supportive, kept them going. Respondents explained that they relied more on their team for support and this helped in ensuring seamless service delivery. HWs reported good interpersonal relationships among themselves which acted positively towards retention.

*My co-workers are supportive, we are colleagues at work. We have good team work [...] Male* Laboratory Assistant, northern Uganda R4

A doctor and manager explained it as ‘a psychological contract’ with the team. He explained that this kept him in the hospital amidst poor salary, and lack of transparency in the organization;

*I have a psychological contract with the team whereby we work well and achieve a lot. Male doctor, eastern region* R27

Another doctor from western Uganda, also explained:

*We work as a team and to me that is an important aspect. […] Teamwork and respect for each other […] Male* Doctor, western Uganda R12

### Dissonance on workload intensity- “*as time goes on you can see that the staff are lazy”*

While the health facilities presented HWs with opportunities for varied experience, the issue of high workload in PNFP health facilities was reported as a major characteristic of the facilities that negatively affected HWs’ retention. HWs explained that the workload affects their work and life balance. However, there was a divergence of views between managers and HWs in relation to workload. Managers explained that complacency increased over time and when tried to demand results, the HWs saw them as being harsh. While HWs indicate that this is significant factor affecting their retention, managers feel that they are simply delivering their outputs within the normal frame of work.

*They are satisfied, but as time goes on you can see that the staff are lazy and not performing as expected, and when they are still new they perform all their duties very well, but as time goes on, they are used to the place and they no longer fear being dismissed.* Female manager, central Uganda R19

Managers also mentioned that HWs needed to be supervised closely so that they could provide good service to patients who pay user fees. The reason why they lose HWs to government hospitals is because of this characteristically strict supervision, which is in contrast to the prevailing lack of supervision in government hospitals*.*

*In a PNFP setting we offer services at a fee, in government setting, services are “free”. So, where someone is paying for a service, and also of cause if you want to achieve delivery of good services we have to make staff work hard_ […] people expect that they are going to get a good service quality service and you cannot get that unless there is supervision. So, the biggest factor why people leave PNFP health facilities to go to government, is because there [government] supervision is less. They do not mind, it doesn’t matter whether you come and work for two hours, as long as you show up at the facility. This is not the case with our facility.* Male Doctor, western Uganda R15

### Non-responsiveness to staff needs- *“work and work, whether your back is breaking!”*

Related to the issue of high workload, HWs also highlighted that facility managers did not care about their well-being and instead only focused on ensuring that the work was done. Some HWs also lamented that there is a tendency of some managers caring about what happens to patients much more than they care about the staff welfare.

*[…] you are expected to work and work and work, whether your back is breaking, you should work and finish the queue of patients (laughter). […] If my director comes and finds me with a patient, he would want to see me clearing the line than knowing whether I have headache or tooth-ache. So that’s the difference. […] Patient, patient, patient how about the people who are looking after the patient? Male* Clinical officer, northern Uganda. R1

Some HWs linked the lack of career development to their managers’ desire to see the HWs working to deliver services, and felt that managers did not prioritize HWs’ career development through further studies, which was negative for retention.

*Whenever you like to get some support, let’s say you want to go for further education or you want to go for a study leave, all they care for is you working. Female* Doctor, central Uganda R21

### Professional and social divides

The importance of congruence in mission and behaviour, including in equity-based decision-making, performance management, participative management and facility governance and management was cited as a major source of tension by HWs. This reflected the basic assumptions and values that influenced behaviour of the HWs in the PNFP health facilities.

### Perceived discrepancy of mission versus staff treatment

The study revealed that there was a discrepancy between the mission and staff treatment that led to de-motivation. This inequity in incentives and access to continuing professional development opportunities cited by the HWs was interpreted as being incompatible with the values of the faith-based PNFPs. These values had a greater appeal for retention of HWs in this study and their absence resulted in HWs’ dissatisfaction. In fact, some of the participants lamented that this is something intolerable that should not be happening in faith based institutions. There is incongruity with the values of the religions that form a basis for these organizations.

*[…] That is why it is better for me to experience this injustice in a different environment, not the church institutions. Because outside you would really accept the fact that, that is how people are, but when you see this happening in a church institution you feel unhappy... M*ale laboratory technologist, western Uganda R3

A nurse working in a northern Uganda hospital narrated how at her hospital, selection of workers to participate in trainings is discriminatory, favouring the staff that studied in a specific PNFP training school attached to the hospital. She explained that this is the main cause of dissatisfaction with that hospital, which is forcing her to search for a new job. The hospital administrators perceived staff trained in their PNFP training schools to be better qualified and hence preferred them in terms of offering job and career development opportunities:

*They pick their students because there is also some training school attached to this hospital. […].* Y*ou see, what does not satisfy me most, it’s that segregation. […] I don’t like that*. *That’s why you find that someone only works for six months and leaves, because of this kind of thing […] So when you don’t qualify from their training school they see you as an incompetent nurse, so I would rather go for a job where they don’t undermine me that I am incompetent. Female* Nurse, northern Uganda, R16

The discrepancy between the mission and staff treatment was also cited in continuing professional development opportunities availed to staff, where participants highlighted that the process for selection of the participants to take up those opportunities is not always seen as fair. Others explained that training opportunities that attract allowances are sensitive and are usually distributed unevenly, that is, if they are distributed at all. In some instances, the supervisors were the ones who attended all training opportunities that involve per-diem and travel, even when they were not best suited for the training.

*The trainings where there is really money, they are always very sensitive I cannot deny that. When they hear that they will be given some allowance, per-diem some administrators really want to be there themselves. And yet, when there is something everyone should benefit*. Female Nurse, northern Uganda R9

There was agreement on most of the factors affecting retention from both the managers’ and HWs’ perspectives. However, issues related to leadership, governance, and workload, only emerged from the interviews with HWs. This points to the fact that the managers may lack awareness, or are simply unwilling to reveal these practices and their influence on HW retention.

### Non-participative management- *“They should not just decide for them or decide for us”*

Participants expressed the view that in some PNFPs there is no consultation of staff on key decisions affecting them, which affects the implementation of these decisions. This top-down approach to leadership was highlighted as affecting staff retention in the health facilities.

*There are certain decisions they make without even consulting people who are implementing the service […] That hurts because we are adults, we must sit and plan for these patients […]. They should not just decide for them or decide for us. We know them better than they do. Female* Nurse, central Uganda R17

*A few members of the management team contribute to dissatisfaction. For example, you may want to discuss some issues, but they see you as someone who is negative […] This is probably because also the managers are also not trained managers, they are medical people, they have not been trained as managers.* Female Doctor, central Uganda R21

The perspective of managers on this issue was different, and did not necessarily concur with the HWs’ perspectives: They mentioned open communication as a practice, which they suggested was useful in preventing conflicts and creating a conducive working environment for staff, positively influencing retention. Some of the communication strategies adopted included regular daily and monthly meetings, and having staff representatives in the top management.

*[…] sometimes we can also call the staff to the office if there are some issues. Also, the staff can also come to us and put their views of what they want to be happening and sometimes we have the general staff meetings and there is openness.* Female Manager, central Uganda R19

### Poor performance management: The *“red ink of condemnation”*

The narratives by HWs also revealed poor performance management within this hierarchical culture in some of the health facilities, especially in the northern and western regions. Some nurses from the northern region said they felt harassed by their managers:

*The bad thing they come and harass* [you] […] *they come “who does this thing, it’s not right, you know, you go that side you, come this side (in a tough voice)”, you can confuse someone* […]*. When you have done something wrong and you have to correct it, instead of calling you, they decide to follow you, instead they start moving around, “this girl, this person has done this and you know”_[...] that kind of thing. I see people following you every time they are checking you even in the ward and again it’s a problem, so people don’t like that*. Female Nurse northern region R16

In one hospital in western Uganda, HWs were required to log in and out, and failure to do so resulted in one’s name being highlighted with red ink, without trying to find out their reasons for being late. The HWs felt ‘condemned’ because of the use of the red ink.

*They also now have a reporting book for clocking in and it is like they are forcing people. If you don’t write in that book you find your name, moreover a professional, written in red ink with five question marks. What is that? Are you the one who paid school fees for me that you can reduce me like that? Red ink! The ink of condemnation! Why can’t they just call the person? […] It is not a bad policy but the way it is handled is the one which is bad and demoralizing- a red pen*? Male laboratory technologist, western Uganda. R3

This resulted in what should be a performance management tool, having a perverse effect on the HWs’ sense of professionalism. Similarly, other HWs also had concerns in relation to poor supportive supervision and lack of recognition. Participants noted that some of their supervisors were dictatorial and did not listen to them, only concentrating on their weaknesses and mistakes and ignoring their better performance, which contributed to staff dissatisfaction and de-motivation, affecting their retention.

*Many people do good work for the hospital but they don’t recognize them, that’s what I see. I mean instead they see the bad part of you […] I’ve never seen anyone being appreciated that this person has done a very good work since I joined this place and that for sure de-motivates staff*. Female Nurse, northern Uganda R16

In some cases, managers overpromised HWs rewards but did not honour their promises, leading to dissatisfaction.

*You find that you sacrifice to work a lot, but then you find that you are not being appreciated. […] There was a time there was an outbreak in this place of haemorrhagic fever […] You know haemorrhagic fever is something so dangerous and they told us they were going to compensate […] But afterwards up to now, they did not give us anything* […] *I felt it was not fair. If Ebola outbreak is to come, who will sacrifice to come? We are not satisfied really […]* Female Nurse, northern Uganda. R9

### “Politics with a small ‘p’”

Participants made references to the political agendas and control of specific actors within the PNFPs, which one participant referred to as ‘the politics’ with a small ‘p’. This took on the form of interference from religious leaders in the day to day running of the health facilities, and putting religious affiliation ahead of service delivery in decision-making, and in-fighting among boards of trustees, governors, managers and administrators. The politics left the staff in a state where they did not know whose instructions to follow and also affected the operation of the facilities as policies were not passed or implemented smoothly. Participants narrated how the bishops, reverends, nuns and brothers (religious leaders) influenced the running of PNFP facilities, sometimes getting into disagreements with the management. A doctor working in the central region explained that this ‘politics’ confirms the saying that “*when two elephants fight, it’s the grass that suffers*” because mostly the HWs are the ones that bear the burden of unfavourable working conditions.

*There is control by the bishops interfering with the management of the hospital. […] You know the power struggle, the bishop wants his influence to be felt, the medical superintendent also wants his influence to be felt. This is in the hospital! […] we know for a fact is that many hospitals struggle, the influence of the church leadership interfering with the hospital management creates a very, very big de-motivating factor […]* Male Medical doctor, central region R11

In one case, a doctor of one church health facility was thought to have acted out of order for partnering with a facility run by another church denomination facility to deliver health services in the catchment area. This was so despite having been discussed with the director and agreeing to the funders’ objectives. Other HWs explained that in some of the PNFP facilities the line between governance and management was blurred. A medical doctor narrated how the organization he was working in was operating without proper management systems, with the founding director as the ultimate authority.

*What I didn’t like I can say the way the director was running the place […] he was running it around himself and there were no functional systems such as a management team and board. You know the way he was running it the way, you would run your home. He was the overall person and that is where I found a problem in the, basically, governance and management.* Male Doctor, central Uganda R11

There were also some tensions revealed between the heavily resourced HIV department and the general hospitals. A doctor in one of the hospitals in central Uganda explained how hospital ‘politics” de-motivates some HWs. This politics is about trying to even out salaries for both those working in the heavily resourced HIV clinics and those in the general hospital side in the same health facility.

*I would call it the politics in the hospital. […] The hospital does not want any differences in salaries between staff in the HIV clinic and the hospital- they must be the same and that is one of the reasons why, some people may also go. Donors as you know in Uganda have really supported HIV care […] So, the staffs working in the HIV clinics, even outside this hospital, usually earn more than other hospital staff. The hospital would not accept that difference, so, comparing salaries of other people working in HIV, this de-motivates people who work in the HIV clinic, so they decide to leave.* Female Doctor, central region R21

Another scenario of work politics was shared by a health worker who was working in the HIV clinic section of the hospital but felt that HWs in this clinic were not recognized by staff working in the general hospital. The root cause of the problem is the politics between the administrators in the HIV clinic and those of the general hospital:

*I would say to an extent there are some political issues in the hospital between the HIV clinic and the hospital […] so we are not recognized I would say. It is between the administration of this side and that side so in the HIV clinic we work but not so recognized that we are doing work in the clinic. […] Is it because we are different from other health workers?* Female Nurse, central region R17

## Discussion

The study confirms the view that culture plays an important role in HWs’ retention and that feelings towards the organization are influenced by HWs’ perceptions. Firstly, the narratives of HWs and managers demonstrated PNFP organizational culture is largely influenced by the work environment, which is part of the organizational structure and processes classified as the visible artefacts. The influence of this on retention was mixed, with HWs’ being positively inclined towards professionalism and self-actualization and good interpersonal relationships while at the same time expressing negative feelings towards the perceived high workload.

The findings indicate that HWs reported that in general the health facilities had good infrastructure and work tools that promoted a positive working environment, professionalism and self-actualization and their retention. This is in contrast to some public-sector facilities in the country that are in need of renovation and suffer from persistent stock-outs of drugs and essential reagents [24, 25]. This affirms findings that having disposal of equipment and supplies has also been reported to improve morale of HWs [26]. The findings also showed that HWs have good relationships among themselves, although there is a considerable distance between HWs and managers in the PNFP facilities. The findings also indicate that interpersonal relationships are an important retention factor in the health facilities. HWs in the facilities may be fostering teamwork as a way of coping with poor organizational culture. Similar findings have been reported in Kenya [27, 28]. This is synonymous with findings that younger workers prefer solid leadership with clear directives [29]. Establishing ties and friendship in a work environment encourages HWs to remain in the health facilities to maintain the social ties, although in some cases HWs reported that there was a clash with experienced values. In West Nile region of Uganda, one of the important causes of attrition was found to be poor working relationships between HWs and managers, while laboratory staff also cited disrespect and lack of recognition by other HWs as a cause of dissatisfaction [25, 30]. The influence of interpersonal relationships on retention has also been found in a study conducted among nurses in South Africa where support from peers, friendship and respect were highlighted in decisions to stay in the workplace [31]. Managers mentioned open communication as a practice, although this did not concur with the HWs’ perspectives. The complex activities in a healthcare setting requires that the different unit managers can work seamlessly with good communication and collaboration [32].

On the other hand, the HWs reported that the high workload affected their job satisfaction, which in turn negatively influences retention. High workload was reported by HWs of all ages and in all regions and almost universally by those working in the northern region. In keeping with the findings, in post-conflict setting HWs were found to usually experience high workload [33]. It does appear that working in PNFPs is synonymous with high workload, that is often perceived as not being commensurate with the rewards, and yet dual practice is also prohibited. Workload was consistently cited by HWs as a factor that influences their retention although none of the managers acknowledged this as a barrier to retention. This divergence may reveal that managers do not actually believe that the workload is high or that effective communication appears to be lacking between HWs and managers in PNFP health facilities. Workload has been cited as a factor affecting retention of HWs in South Africa and the Eastern Mediterranean region [31, 34]. Additionally, the HWs suggested that there was need to strike a balance between being externally and internally focused. Faith-based PNFP health facilities in Uganda are motivated by their religious beliefs to treat the sick and as a result they value the trust of their patients and do not want to compromise on this [7]. This study has confirmed this, but more so has shown that HWs believe that PNFPs could also be equally internally focused, including offering support or assistance when HWs experienced personal problems.

Secondly, the findings revealed that there were professional and social divides that were a source of tension between the HWs and the facility management. These tensions reflect the basic assumptions and values that influenced behaviour of the HWs in the PNFP health facilities and were characterized by negative emotions expressed towards the lack of equity in opportunities, non-participative management, poor performance management and tensions in governance and management. There was some incongruity in ethical values in relation to equity and fairness that resulted in HWs’ dissatisfaction. Inequity in incentives and access to continuing professional development opportunities was cited by the HWs as damaging for retention. Values that are related to fair and just treatment of HWs had a greater appeal for retention of HWs in this study. Participants indicated that they work outside their schedules and some of this work is not recognised and managers do not keep their promises. Recognition of HWs by managers is important so that they are motivated to continue working in the health facilities. The study has shown that in general while managers reported open communication, this was not actually congruent with what the HWs reported. These findings concur with the application of the equity theory which postulates that employees tend to compare the way they are treated with others in the same organization and if they feel that they are being treated unfairly, their work effort decreases [35]. In this case, this extended further to comparing the organizational behaviour to its ethos. In addition, to the shared values, HWs in PNFPs expect fair treatment and an environment of trust [36]. For the faith-based PNFPs, grounded in strong religious philosophies, there is need for sustained engagement and working around their beliefs and values while at the same time working with HWs to help them to advocate and package their concerns in a non-threatening manner.

HWs felt that their managers did not practice a participative management style coupled with poor performance management. Decisions were imposed on them, with one-directional top-down communication, and disempowering HWs from participating or shaping the decisions at the health facilities, coupled with poor performance management which affected on their professionalism, leaving them feeling incompetent and infantilized. In the public sector in Uganda, there are also reports of inadequate performance management of HWs [37]. Similarly, a study in the public sector in Sierra Leone and found that some junior cadres felt unappreciated in their work [23]. This resulted in tensions in the workplace, suggesting some similarities with our study in the PNFP sub-sector in Uganda. Managers used their positional power to make HWs feel that their contributions were not valued and also made themselves unapproachable by not addressing staff concerns. This resulted in reduced motivation and negatively affected retention. This may be attributed to the strong, authoritarian and unquestionable nature of religious and political authority in the country (most facilities are faith-based but there are also some founded by political or community leaders who wield a lot of power). This may be noticeable in aspects of organizational structure, decision making and policy content. The findings in the study underscore the relevance of good leadership and management, where HWs are consulted or involved in decision making, recognized and supervised in a supportive manner to ensure staff retention in PNFP organizations and presumably other settings too. The findings from this study concur with other studies that suggest that participative management style could lead to better retention [38, 39]. A study in Kenya revealed that HWs’ motivation was higher where the leadership was strong and supportive [27].

The study illuminated the tensions between HWs, managers and religious motivations and values in PNFP health facilities and the influence on retention of HWs. While not widespread, there were instances when the need to put religious affiliation or mission at the forefront of the service appeared to conflict with the ethos of service delivery or professionalism of HWs. Some alliances with health facilities from other denominations were viewed as a risk, potentially leading to the erosion of the sense of mission or identity of the health facilities. In this sense religion may be a barrier to professionalism, negatively affecting motivation and retention of HWs. This may be conflicting with the message that faith-based PNFPs provide service without discrimination, as in asserting their identities, they can also lead to exclusion. Similarly, in public sector facilities in West Nile region in Uganda, the major reason why HWs left was political interference, in this case by political leaders [30]. Our findings also concur with findings from the cross-sectional survey conducted in Mahalapye and Ngamiland districts of Botswana which found that the most important aspects of the organizational culture affecting motivation of HWs were the basic assumptions and values, including transparency, professional growth, staff recognition, shared decision-making, leadership development and teamwork [22].

### Strengths and limitations

The link between organizational culture and retention in the PNFP sub-sector in Uganda has been largely unexplored, and indeed across Africa the number of studies which have examined this issue in depth is limited. This study has provided empirical evidence on the influence of perceived organizational culture on retention within the PNFP context. The evidence in this study is self-reported, which means that what HWs say about desire to stay may not be reflected ultimately in their behaviour. During the recruitment of participants, the reliance on volunteers could have resulted in selection bias [40, 41] and while various cadres from the different regions were interviewed, they may not represent the wide range of opinions among HWs. The volunteers may also have had certain characteristics that could have biased the findings. However, the use of qualitative methods provides a rich description of the perspectives and the way this culture is experienced. The design of the study was such that it only focused on the PNFP sector, with the majority of them being faith-based and care should be taken not to generalize to non-faith-based PNFPs and the public sector, which has different a management system and organization. Care must be taken in generalizing the findings to the entire PNFP sector in Uganda as the study health facilities might have also had some unique characteristics which could differ from other PNFP hospitals. More research is needed to examine successful strategies in improving organizational culture in PNFP facilities, including promoting participative leadership and management.

## Conclusion

Private-not-for-profit health facilities have a service ethic, which is part of the visible artefacts which means that they also provide infrastructure and work tools that enable HWs to provide services effectively. On the other hand, however, high workload and lack of staff-centeredness negatively influenced retention. In terms of basic assumption and values, the PNFP facilities were characterised by actions that were not congruent with their values including inequity in staff treatment, non-participative management, poor performance management and tensions in governance and management. Nonetheless, HWs reported good interpersonal relationships that helped them to navigate their roles within the context of non-participative management. While not widespread, there were instances when the need to put religious affiliation or mission at the forefront of the service appeared to conflict with the ethos of service delivery or professionalism of HWs. Fostering a more inclusive and open culture requires a more participative and transformational leadership style.

Our findings have implications for improving retention. The organizational culture must be considered as an important factor which influences HWs’ retention. Managers of PNFP health facilities should periodically conduct organizational culture assessments, evaluate how they influence outcomes such as retention and develop appropriate strategies. To help to facilitate this process of transformation, it will be important to engage external facilitators who are neutral to help the health facilities reflect on their organizational culture. There is need to create positive enabling environments, where HWs can work with managers in building collective confidence and shaping potential retention strategies, with effective communication through an open and reflective process. The culture and hence expectations regarding HWs’ behaviour in the PNFP facilities appeared to be deeply rooted in the historical religious patterns and traditions related to the founding values and may therefore be hard to shift.
